# Direct and Indirect Effects of SARS-CoV-2 Pandemic in Subjects with Familial Hypercholesterolemia: A Single Lipid-Center Real-World Evaluation

**DOI:** 10.3390/jcm10194363

**Published:** 2021-09-24

**Authors:** Roberto Scicali, Salvatore Piro, Viviana Ferrara, Stefania Di Mauro, Agnese Filippello, Alessandra Scamporrino, Marcello Romano, Francesco Purrello, Antonino Di Pino

**Affiliations:** 1 Department of Clinical and Experimental Medicine, University of Catania, 95100 Catania, Italy; spiro@unict.it (S.P.); vivi.fer@hotmail.it (V.F.); 8stefaniadimauro6@gmail.com (S.D.M.); agnese.filippello@gmail.com (A.F.); alessandraska@hotmail.com (A.S.); francesco.purrello@unict.it (F.P.); nino_dipino@hotmail.com (A.D.P.); 2 Geriatric Unit, Garibaldi Hospital, 95100 Catania, Italy; marcelloromanoct@gmail.com

**Keywords:** SARS-CoV-2 pandemic, familial hypercholesterolemia, lipid-lowering therapy, healthcare system, cardiovascular risk

## Abstract

We evaluated the impact of direct and indirect effects of SARS-CoV-2 infection in subjects with familial hypercholesterolemia (FH). In this observational, retrospective study, 260 FH subjects participated in a telephone survey concerning lipid profile values, lipidologist and cardiologist consultations and vascular imaging evaluation during the 12 months before and after the Italian lockdown. The direct effect was defined as SARS-CoV-2 infection; the indirect effect was defined as the difference in one of the parameters evaluated by the telephone survey before and after lockdown. Among FH subjects, the percentage of the lipid profile evaluation was lower after lockdown than before lockdown (56.5% vs. 100.0%, *p* < 0.01), HDL-C was significantly reduced (47.78 ± 10.12 vs. 53.2 ± 10.38 mg/dL, *p* < 0.05) and a significant increase in non-HDL-C was found (117.24 ± 18.83 vs. 133.09 ± 19.01 mg/dL, *p* < 0.05). The proportions of lipidologist and/or cardiologist consultations and/or vascular imaging were lower after lockdown than before lockdown (for lipidologist consultation 33.5% vs. 100.0%, *p* < 0.001; for cardiologist consultation 22.3% vs. 60.8%, *p* < 0.01; for vascular imaging 19.6% vs. 100.0%, *p* < 0.001); the main cause of missed lipid profile analysis and/or healthcare consultation was the fear of SARS-CoV-2 contagion. The percentage of FH subjects affected by SARS-CoV-2 was 7.3%. In conclusion, a lower percentage of FH subjects underwent a lipid profile analysis, lipidologist and cardiologist consultations and vascular imaging evaluation after SARS-CoV-2 Italian lockdown.

## 1. Introduction

Since December 2019, the severe acute respiratory syndrome coronavirus 2 (SARS-CoV-2) pandemic has affected more than 190,000,000 subjects and caused more than 4,000,000 deaths worldwide [1]. The clinical manifestations of SARS-CoV-2 infection broadly differ among the affected subjects; about half of the infected subjects remain asymptomatic, the majority of the symptomatic subjects experience influenza-like symptoms and 10–15% of these develop a severe disease (COVID-19) characterized by a wide clinical scenario from pneumonia to acute respiratory distress syndrome (ARDS) and disseminated intravascular coagulation [2].

Other than the respiratory tract, COVID-19 can also affect the cardiovascular system. In fact, several mechanisms of SARS-CoV-2 heart injury have been hypothesized: direct myocardial damage by binding the SARS-CoV-2 spike glycoprotein to angiotensin-converting enzyme 2, cardiac inflammation in the context of cytokine release syndrome (cytokine storm) caused by progression of COVID-19, increased myocardial distress in the context of ARDS and coronary plaque rupture due to increased endothelial shear stress [3].

Beyond the reported direct damage of SARS-CoV-2 infection, increasing attention has been focused on the indirect effects of the SARS-CoV-2 pandemic because of the healthcare public system restructuring; in particular, a substantial reduction in hospital admissions for acute coronary syndromes (ACS) during the SARS-CoV-2 pandemic was shown related to the national lockdown in Italy, and this could be explained by increasing fear of in-hospital contagion, an emergency department overload and the healthcare structure remodeling [4]. In this context, the reduced cardiovascular screening may be deleterious in subjects at high cardiovascular risk such as those with familial hypercholesterolemia (FH), which is the most frequent monogenic disorder characterized by a lifelong elevation of low-density lipoprotein cholesterol (LDL-C) and early atherosclerotic cardiovascular disease (ASCVD) [5]. Thus, the delay of clinical and/or genetic diagnosis and the deferred lipid-lowering therapy optimization could promote an increase in LDL-C burden strongly associated with atherosclerotic injury progression [6,7,8].

In this study, we evaluated the direct and indirect effects of the SARS-CoV-2 pandemic in a cohort of FH subjects.

## 2. Methods

### 2.1. Study Design and Population

This was a retrospective, observational study involving patients aged over 18 years with a genetically confirmed FH diagnosis [9] and enrolled from the Lipid Centre of the University Hospital of Catania, Italy, from 4 June 2021 to 9 August 2021. All participants had a telephone survey concerning their lipid profile values (total cholesterol, high-density lipoprotein cholesterol (HDL-C), triglycerides (TG), LDL-C), lipidologist and cardiologist consultations, vascular imaging evaluation and lipid-lowering therapy adherence in the 12 months before and after the Italian lockdown (9 March–3 June 2020); moreover, all participants confirmed or not the SARS-CoV-2 infection from 9 March 2020 to 12 months after the end of the Italian lockdown (3 June 2020). Vascular imaging was defined by carotid and/or femoral ultrasound evaluation. Statin therapy was divided into three categories according to the efficacy of LDL-C reduction: high intensity (≥50% LDL-C reduction, rosuvastatin 20–40 mg/day or atorvastatin 40–80 mg/day), moderate intensity (30–50% LDL-C reduction, rosuvastatin 5–10 mg/day, atorvastatin 10–20 mg/day, simvastatin 20–40-80 mg/day, pravastatin 40 mg/day, fluvastatin 80 mg/day, lovastatin 40 mg/day) or low intensity (<30% LDL-C reduction, simvastatin 10 mg, pravastatin 20 mg/day, fluvastatin 40 mg/day, lovastatin 20 mg/day). Type 2 diabetes and arterial hypertension were defined as the daily intake of glucose-lowering medication and antihypertensive drugs, respectively. ASCVD and LDL-C targets were defined as previously described [10]. Long-wait consultation was defined as >3 months. Hospitalizations for COVID or other comorbidities were also reported. The SARS-CoV-2 pandemic direct effect was defined as the virus-related infection from 9 March 2020 to 12 months after the end of the Italian lockdown. The SARS-CoV-2 pandemic indirect effect was defined as the difference in one of the following evaluated parameters in the 12 months before and after the Italian lockdown: lipid profile analysis, lipidologist and cardiologist consultations and vascular imaging evaluation.

### 2.2. Statistical Analysis

The distributional characteristics of each variable, including normality, were assessed by the Kolmogorov–Smirnov test. Data are reported as mean ± standard deviation (SD) for continuous parametric parameters, median (interquartile range (IQR)) for continuous nonparametric variables and frequency (percentage) for categorical variables. When necessary, the continuous nonparametric variable “TG” was logarithmically transformed to reduce skewness. To test differences in clinical and biochemical characteristics of the study population before and after Italian lockdown, we used Student’s t-test. The χ^2^ test was used for categorical variables. All statistical analyses were performed using IBM SPSS Statistics for Windows version 23. For all tests, *p* < 0.05 was considered significant.

The study was approved by the local ethics committee (prot. number 46/19) in accordance with the ethical standards of the institutional and national research committees and with the 1964 Declaration of Helsinki and its later amendments or comparable ethical standards.

Informed consent was obtained from each subject enrolled in the study.

## 3. Results

**Figure 1:**
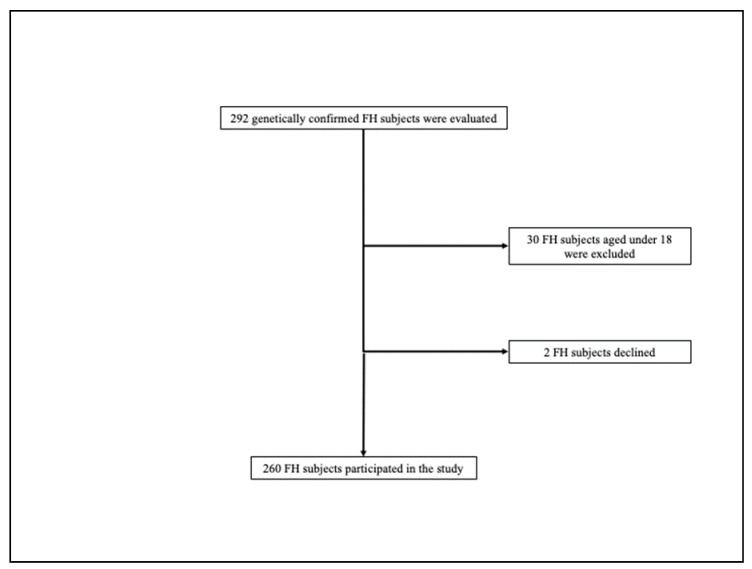
Enrollment flowchart of the study population. FH = familial hypercholesterolemia.

In total, 292 genetically confirmed FH subjects were evaluated; of these, 30 subjects did not satisfy the inclusion criteria and 2 subjects declined. Finally, 260 FH subjects participated in the study (Figure 1).

Table 1 shows the baseline characteristics of the study population; 49.6% of FH subjects were males, and the percentage of subjects with a history of ASCVD was 30.8%. The majority of FH subjects exhibited a pathogenic variant in the LDL receptor (LDLR), and 97.7% of subjects were heterozygotes; three subjects were double heterozygotes, two subjects were compound heterozygotes and one subject was homozygote. Concerning the presence of cardiovascular risk factors, the percentage of diabetic FH subjects was 2.3%, 27.7% of subjects were hypertensive and 22.7% of subjects were smokers; the proportion of FH subjects with at least two of the mentioned risk factors was 13.1%. Concerning lipid-lowering treatments, the majority of FH subjects were on statins; in particular, 73.3% of subjects took high-intensity statins, 24.6% of subjects were on moderate-intensity statins and only 1.9% of subjects were statin-intolerant. Furthermore, the percentage of FH subjects on ezetimibe was 86.5%, and 23.8% of subjects were on PCSK9-i therapy; finally, the proportion of subjects on statin and ezetimibe and PCSK9-i was 21.9%.

The direct and indirect effects of SARS-CoV-2 are reported in Table 2. Among FH subjects, the percentage of the lipid profile evaluation was lower after lockdown than before lockdown (56.5% vs. 100.0%, *p* < 0.01); moreover, HDL-C was significantly reduced after lockdown compared to before lockdown (47.78 ± 10.12 vs. 53.2 ± 10.38 mg/dL, *p* < 0.05), and a significant increase in non-HDL-C was found after lockdown compared to before lockdown (117.24 ± 18.83 vs. 133.09 ± 19.01 mg/dL, *p* < 0.05).

**Figure 2:**
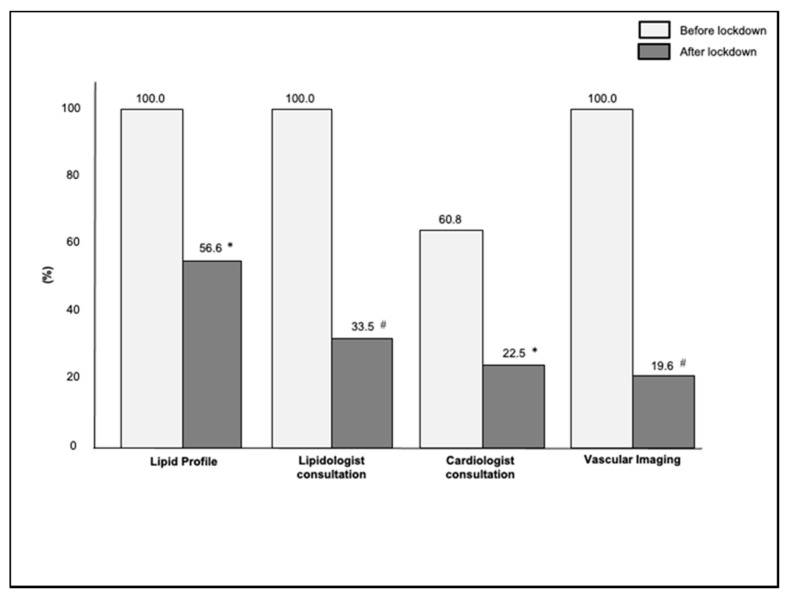
Percentages of lipid profile analysis, lipidologist and cardiologist consultations and vascular imaging evaluation in the study population. * *p* value < 0.01 vs. before lockdown, ^#^ *p* value < 0.001 vs. before lockdown.

The proportion of FH subjects who had lipidologist and/or cardiologist consultations and/or vascular imaging was lower after lockdown than before lockdown (for lipidologist consultation 33.5% vs. 100.0%, *p* < 0.001; for cardiologist consultation 22.3% vs. 60.8%, *p* < 0.01; for vascular imaging 19.6% vs. 100.0%, *p* < 0.001) (Figure 2); the main cause of missed lipid profile analysis and/or healthcare consultations was the fear of contagion. Finally, the percentage of FH subjects affected by SARS-CoV-2 was 7.3%, and none of them required hospitalization.

As concerns the FH subjects who reported having contracted SARS-CoV-2 infection (Table 3), the mean age was 58.7 ± 5.18, 52.6% of subjects were males and the proportion of subjects with a history of ASCVD was 78.9%. While the majority of SARS-CoV-2-affected FH subjects were on intensive lipid-lowering therapies, only 42.1% of subjects achieved the LDL-C target according to the European Society of Cardiology/European Atherosclerosis Society Guidelines 2019 for the management of dyslipidemias. Concerning the cardiovascular risk factors, 15.8% of FH subjects were diabetics, 84.2% of subjects were hypertensive and 36.8% of subjects were smokers; the percentage of FH subjects with at least two of the mentioned risk factors was 52.6%. Finally, the majority of FH subjects were on high-intensity statins, all subjects took ezetimibe and the proportion of subjects on statins plus ezetimibe plus PCSK9-i was 63.2%.

## 4. Discussion

Over the last year, increasing attention has been focused on the direct effects of the SARS-CoV-2 pandemic such as the prevalence of infection, COVID-19, hospitalization and death and its indirect effect related to the impact of SARS-CoV-2 pandemic on the healthcare system. In this retrospective observational study, we evaluated the impact of direct and indirect effects of SARS-CoV-2 pandemic in a cohort of subjects at high cardiovascular risk; to the best of our knowledge, this is the first study focusing on SARS-CoV-2 pandemic impact in this population. We found that a lower percentage of FH subjects underwent lipid profile evaluation after the SARS-CoV-2 Italian lockdown; furthermore, a reduction in HDL-C and an increase in non-HDL-C were observed in FH subjects after lockdown. In this context, a hypothetical explanation of these findings could be a dysregulated lifestyle including reduced physical activity and a high-fat diet during the SARS-CoV-2 pandemic; in line with this hypothesis, previous findings showed that reduced physical activity and an increase in BMI were two main effects of the SARS-CoV-2 lockdown [11,12].

In our study, we found that a lower proportion of FH subjects received lipidologist and cardiologist consultations and vascular imaging evaluation after the SARS-CoV-2 lockdown; the main explanation of these findings obtained from FH subjects by telephone survey was the fear of SARS-CoV-2 contagion. This finding was in line with two previous findings that evaluated the impact of SARS-CoV-2 infection on the healthcare system. In fact, Cori et al. showed in the EPICOVID19 web-based Italian survey that 65% of subjects reported fear of SARS-CoV-2 contagion for themselves and family members [13]; moreover, Amorim et al. reported that the admission of patients with ST-elevation myocardial infarction was significantly reduced in the emergency department during SARS-CoV-2 lockdown [14]. Taking into these findings, our study highlighted that the indirect effects of the SARS-CoV-2 pandemic could be deleterious in the cardiovascular risk management of FH subjects; future prospective studies are needed to evaluate the prognostic role of our findings.

In our study, the percentage of FH subjects with SARS-CoV-2 infection was 7.3%, in line with Italian SARS-CoV-2 prevalence [15]; in this context, it could be hypothesized that the risk of SARS-CoV-2 infection was similar between FH subjects and the general population. Moreover, we found that SARS-CoV-2-affected FH subjects had a BMI and age over the mean of the study population, and the majority of them had a prior ASCVD. Furthermore, SARS-CoV-2-affected FH subjects had a before-lockdown HDL-C under the mean of the study population; thus, it could be hypothesized that a low HDL-C level could increase the risk of SARS-CoV-2 infection. In line with this hypothesis, Hilser et al. found that a 10 mg/dL increase in HDL-C or apolipoprotein AI was associated with a 10% reduction in risk of SARS-CoV-2 infection [16]. Although the majority of SARS-CoV-2-affected FH subjects were on intensive lipid-lowering therapy, only 40% of them achieved the LDL-C target according to the European Society of Cardiology/European Atherosclerosis Society Guidelines 2019 for the management of dyslipidemias; moreover, more than 50% of SARS-CoV-2-affected FH subjects had two or more cardiovascular risk factors. Thus, it could be hypothesized that an LDL-C beyond the recommended targets in concomitance with other cardiovascular risk factors could increase the risk of SARS-CoV-2 infection in FH subjects. In line with this hypothesis, Lusignan et al. showed in the Oxford Royal College of General Practitioners (RCGP) Research and Surveillance Centre primary care network that subjects with SARS-CoV-2 infection had several cardiovascular risk factors and the presence of diabetes and/or smoking and/or arterial hypertension increased the risk of SARS-CoV-2 infection [17]. Finally, in our study, none of the affected FH subjects required hospitalization; future studies in larger cohorts of FH subjects are needed to confirm and explain this preliminary finding. However, previous findings showed that among subjects with SARS-CoV-2 infection requiring hospitalization, statin users were associated with lower mortality than non-statin users [18,19,20]. Taking these findings into consideration, a possible hypothesis could be that subjects with a long duration of statin therapy, such as FH subjects, could be characterized by a reduced need for hospitalization [21].

There are several limitations to our study. First, this was a retrospective, observational study, and lifestyle evaluation and lipid-lowering therapy adherence were not reported; future prospective studies are needed to correctly evaluate these parameters. Moreover, the study population size was relatively small; for this reason, our preliminary findings should be confirmed in a larger cohort of FH subjects. Finally, a possible pathophysiological link of the atherosclerotic injury in SARS-CoV-2 subjects and FH subjects has not been evaluated; future prospective studies are needed to evaluate this feature.

In conclusion, a lower percentage of FH subjects underwent a lipid profile analysis, lipidologist and cardiologist consultations and vascular imaging evaluation after SARS-CoV-2 lockdown; moreover, reduced HDL-C and increased non-HDL-C were observed in FH subjects after SARS-CoV-2 lockdown, Finally, SARS-CoV-2-affected FH subjects exhibited an LDL-C beyond the recommended targets in concomitance with other cardiovascular risk factors; future prospective studies in a larger cohort of FH subjects are needed to confirm these preliminary findings.

## Figures and Tables

**Table 1:** Baseline characteristics of the study population.

	FH (*n* = 260)
Demographic Characteristics Age, years	49.4 ± 6.22
Men, *n* (%)	129 (49.6)
ASCVD, *n* (%)	80 (30.8)
Body mass index, kg/m^2^	25.3 ± 2.24
FH Genotype	
Pathogenic variants, *n* (%)	267 (100.0)
LDLR, *n* (%)	261 (97.7)
ApoB, *n* (%)	4 (1.5)
PCSK9, *n* (%)	1 (0.4)
ApoE, *n* (%)	1 (0.4)
FH Phenotype	
Heterozygous, *n* (%)	254 (97.7)
Double heterozygous, *n* (%)	3 (1.1)
Compound heterozygous, *n* (%)	2 (0.8)
Homozygous, *n* (%)	1 (0.4)
Pretreated Lipid Profile TC, mg/dL	362.38 ± 19.48
HDL-C, mg/dL	51.38 ± 10.5
TG, mg/dL	96.5 (71.5–115.5)
LDL-C, mg/dL	257.53 ± 18.15
Non-HDL-C, mg/dL	301.51 ± 19.12
Risk Factors Type 2 diabetes, *n* (%)	6 (2.3)
Hypertension, *n* (%)	72 (27.7)
Smokers, *n* (%)	59 (22.7)
≥2 risk factors, *n* (%)	34 (13.1)
Treatments	
High-intensity statin, *n* (%)	191 (73.5)
Moderate-intensity statin, *n* (%)	64 (24.6)
Low-intensity statin, *n* (%)	-
Statin intolerant, *n* (%)	5 (1.9)
Ezetimibe, *n* (%)	225 (86.5)
PCSK9 inhibitor, *n* (%)	62 (23.8)
Statin plus ezetimibe, *n* (%)	195 (75.0)
Statin plus ezetimibe plus PCSK9 inhibitor, *n* (%)	57 (21.9)
Antiplatelet therapy, *n* (%)	80 (30.8)

Data are presented as mean ± standard deviation, percentages or median (interquartile range). FH = familial hypercholesterolemia, ASCVD = atherosclerotic cardiovascular disease, LDLR = low-density lipoprotein receptor, ApoB = apolipoprotein B, PCSK9 = proprotein convertase subtilisin-kexin type 9, ApoE = apolipoprotein E, TC = total cholesterol, HDL-C = high-density lipoprotein cholesterol, TG = triglycerides, LDL-C = low-density lipoprotein cholesterol.

**Table 2:** Direct and indirect effects of SARS-CoV-2 pandemic in the study population.

	FH (*n* = 260) before Lockdown	FH (*n* = 260) after Lockdown	*p* Value
Indirect Effect			
Lipid Profile, *n* (%)	260 (100.0)	147 (56.5)	<0.01
TC, mg/dL *	169.61 ± 18.75	177.83 ± 18.91	0.43
HDL-C, mg/dL *	53.2 ± 10.38	47.78 ± 10.12	<0.05
TG, mg/dL *	90.5 (68.25–114.5)	97.5 (70.5–121.25)	0.11
LDL-C, mg/dL *	103.13 ± 18.02	111.32 ± 18.14	0.25
Non-HDL-C, mg/dL *	117.24 ± 18.83	133.09 ± 19.01	<0.05
LDL-C target, *n* (%) *	105 (40.4)	81 (31.2)	0.09
Lipidologist consultation, *n* (%)	260 (100.0)	87 (33.5)	<0.001
Cardiologist consultation, *n* (%)	158 (60.8)	58 (22.3)	<0.01
Vascular imaging, *n* (%)	260 (100.0)	51 (19.6)	<0.001
Cause of Indirect Effect			
Contagion fear, *n* (%)	-	218 (83.8)	-
Long-wait consultation, *n* (%)	-	42 (16.2)	-
Direct Effect			
SARS-CoV-2 infection, *n* (%)	-	19 (7.3)	-
Hospitalization			
COVID-19, *n* (%)	-	-	-
Other causes, *n* (%)	-	-	-

Data are presented as mean ± standard deviation, percentages, or median (interquartile range). FH = familial hypercholesterolemia, TC = total cholesterol, HDL-C = high-density lipoprotein cholesterol, TG = triglycerides, LDL-C = low-density lipoprotein cholesterol, LLT = lipid-lowering therapy, SARS-CoV-2 = severe acute respiratory syndrome coronavirus 2, COVID = coronavirus disease. * Student’s t-test was performed in subjects for whom the lipid profile was evaluated before and after lockdown.

**Table 3:** Characteristics of FH subjects affected by SARS-CoV-2.

	SARS-CoV-2 FH (*n* = 19)
Demographic Characteristics	
Age, years	58.7 ± 5.18
Male, *n* (%)	10 (52.6)
Body mass index, kg/m^2^	26.1 ± 1.52
ASCVD, *n* (%)	15 (78.9)
FH Phenotype	
Heterozygote, *n* (%)	15 (78.8)
Double heterozygote, *n* (%)	1 (5.3)
Compound heterozygote, *n* (%)	2 (10.6)
Homozygote, *n* (%)	1 (5.3)
Lipid Profile Before Lockdown	
TC, mg/dL	162.45 ± 10.24
HDL-C, mg/dL	49.8 ± 10.13
TG, mg/dL	97.25 (66.0–113.5)
LDL-C, mg/dL	93.34 ± 10.11
Non-HDL-C, mg/dL	113.36 ± 10.43
LDL-C target, *n* (%)	8 (42.1)
Risk Factors	
Type 2 diabetes, *n* (%)	3 (15.8)
Hypertension, *n* (%)	16 (84.2)
Smokers, *n* (%)	7 (36.8)
≥2 risk factors, *n* (%)	10 (52.6)
Treatments	
High-intensity statin, *n* (%)	17 (89.5)
Moderate-intensity statin, *n* (%)	2 (10.5)
Low-intensity statin, *n* (%)	-
Statin intolerant, *n* (%)	-
Ezetimibe, *n* (%)	19 (100)
PCSK9 inhibitor, *n* (%)	12 (63.2)
Statin + ezetimibe + PCSK9 inhibitor, *n* (%)	12 (63.2)
Antiplatelet therapy, *n* (%)	15 (78.9)

Data are presented as mean ± standard deviation, percentages or median (interquartile range). FH = familial hypercholesterolemia, ASCVD = atherosclerotic cardiovascular disease, TC = total cholesterol, HDL-C = high-density lipoprotein cholesterol, TG = triglycerides, LDL-C = low-density lipoprotein cholesterol, PCSK9 = proprotein convertase subtilisin-kexin type 9.
